# Utilizing the micron sized non-thermal atmospheric pressure plasma inside the animal body for the tumor treatment application

**DOI:** 10.1038/srep29048

**Published:** 2016-07-07

**Authors:** Shahriar Mirpour, Somayeh Piroozmand, Neda Soleimani, Neda Jalali Faharani, Hamidreza Ghomi, Hoda Fotovat Eskandari, Ali Mohammad Sharifi, Sahar Mirpour, Mohammad Eftekhari, Maryam Nikkhah

**Affiliations:** 1 Laser and Plasma institute, Shahid Beheshti University, Tehran, Iran; 2 Department of Nanobiotechnology, Faculty of Biological Sciences, Tarbiat Modares University, Tehran, Iran; 3 Department of Microbiology, Faculty of Biological Sciences, Shahid Beheshti University, Tehran, Iran; 4 Plasma Physics Research Center, Science and Research branch of Islamic Azad University, Tehran, Iran; 5 Razi Drug Research Center and Department of Pharmacology, School of Medicine, Iran University of Medical Sciences, Tehran, Iran; 6 Russell H. Morgan Department of Radiology and Radiologic Science, Johns Hopkins University, MD, USA

## Abstract

This study aimed to evaluate the effects of micron sized non-thermal atmospheric pressure plasma inside the animal body on breast cancer tumor. The μ-plasma jet consists of micron sized hollow tube in which pure helium gas is ionized by high voltage (4 kV) and high frequency (6 kHz). The efficiency of the plasma treatment in killing cancer cells was first investigated by cell viability measurements of treated 4T1 cells using flow cytometry and cell cycle analysis. For exploration of the *in vivo* effects of the plasma treatment, the BALB/c mice inoculated by 4T1 cell lines were exposed subcutaneously to plasma for 3 minutes. In addition, H&E staining, TUNEL and Western blotting assays were performed in order to observed the effects of the non-thermal plasma on the tumor cells. The results showed that the efficiency of the plasma in suppression of the tumor growth is comparable to that of a typical chemotherapy drug. Moreover, the results indicated that the plasma induces apoptosis in the tumor tissue and increases the ratio of the apoptotic to anti-apoptotic protein expression. We believe that these findings presented herein may extend our knowledge of the mechanisms by which the plasma exerts its promising anti-cancer effects.

The cancer treatment by non-thermal atmospheric pressure plasma has attracted considerable attentions to the field of cancer therapy by using cold plasma^1^,^2^,^3^,^4^,^5^. The conventional cancer therapy techniques are associated with many issues such as the normal tissue damage, time consuming treatment procedure and expensive therapies^6^. The non-thermal plasma treatment has been introduced as a cost effective, rapid and low damage treatment which may represent an alternative for the conventional methods. Plasma contains the reactive species^7^,^8^, free radicals^9^, energetic ions^10^ and also the transient electric fields inherent with plasma delivery^11^,^12^ which are formed in the atmospheric room temperature medium and interact with the cells and other living organisms. It was shown that the plasma induced the apoptotic cell death in cancer cells while it had no adverse effect on the normal cell lines in the appropriate dosage^13^,^14^. The results also indicated that by exposing the free radicals to cancer cells, the cells could produce the intracellular reactive oxygen species (ROS) which could cause apoptotic cell death^15^. In addition, the role of nitric oxide (NO) generated by plasma is considerable^16^,^17^. Moreover many other studies tried to figure out the mechanism of the cell death in cancer cells treated by the non-thermal plasma such as the cell signals activation induced by the reactive agents^18^,^19^,^20^,^21^. Meanwhile, few studies have presented the effect of non-thermal plasma on the *in-vivo* tumor models^22^,^23^. The reports have shown the tumor suppression with different dosage of the plasma treatment^23^. The researchers also tried to figure out the mechanism of the plasma tumor interaction^23^,^24^. One of the benefits of the plasma treatment in the *in-vivo* model is that the plasma has low harm effects on the normal tissue in the appropriated dosages^25^.

One of the aims of the plasma medicine is to employ the atmospheric plasma jet inside the living creature’s body. The atmospheric pressure plasma is not applicable for cancer therapy in the conventional form, because of some major drawbacks such as the plasma probe volume, high voltage issue, gas delivery and the formation of discharge in the organ. Moreover the heat inside the organ should be controllable and should not harm the normal tissues. Some studies were done to utilize the atmospheric plasma inside the body by miniaturization of the plasma in the micron sized probe^26^,^27^. In addition some other researchers investigated the optimization of the atmospheric pressure plasma devices in the μ-sized shape both in the helium jet and the dielectric barrier discharge forms in order to conduct the plasma species to the living animals^28^,^29^,^30^. Furthermore, the researchers tried to employ the μ-sized plasma jet for a single cell therapy^26^. However, these studies were not completely successful for carrying out the atmospheric plasma treatment inside the animal body.

This study aimed to perform the plasma treatment inside the animal model and to evaluate the efficiency of micron-sized non-thermal atmospheric pressure plasma on the tumor growth suppression. In this regards, followed by the fabrication and characterization of the μ-plasma, the possible mechanisms of the *in vitro* and *in vivo* plasma-cancer cells interactions were further investigated.

## Results

### Plasma characterization

**Figure 1:**
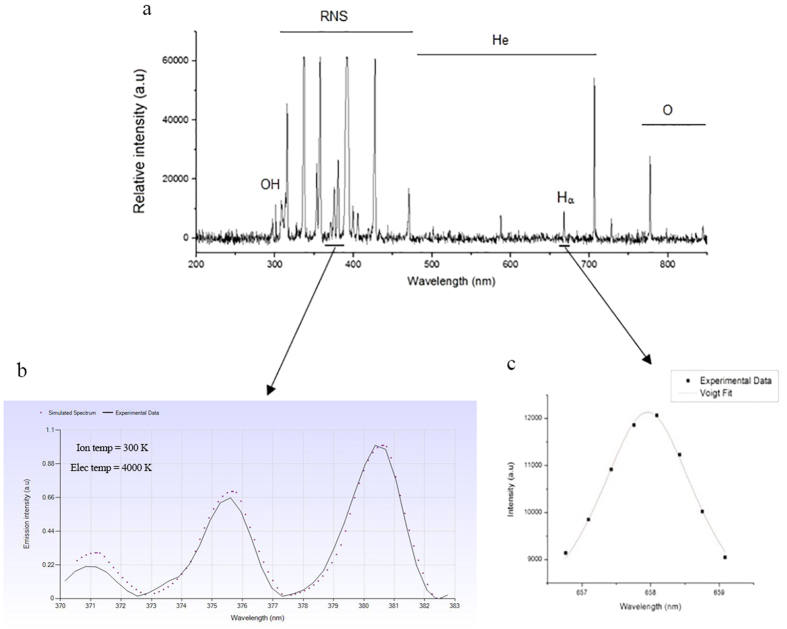
(**a**) Optical emission spectroscopy of the μ-plasma jet in the range of 200–1000 nm. (**b**) Ion and electron temperature analyses of the nitrogen SPS experiment. (**c**) Analyses of the H_α_ transition band for evaluation of the plasma electron density.

Figure 1a represents the species generated in the plasma. Results show that reactive particles of the oxygen atom (777 nm) and OH (310 nm) are of the most important constituents of plasma. From the analyzing of the nitrogen second positive system, ranging from 370 nm to 382 nm, electron temperature of 4000 ± 300 K and ion temperature of 300 ± 5 K were obtained (Fig. 1b). The plasma density was obtained as (4.8 ± 1.2) × 10^13^ 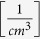 from the analyzing the H_α_ line broadening (Fig. 1c).

### Plasma inhibits the metastasis of the cancer cells

**Figure 2:**
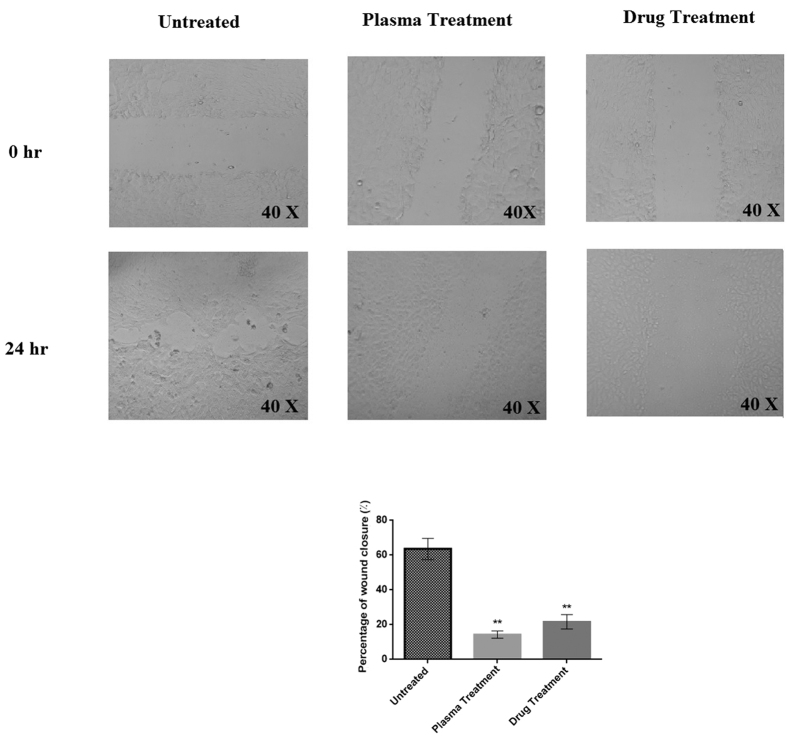
Cell migration assessment by wound healing assay, 24 hours after the plasma and drug treatments. The magnification of the images is 40X. (**P < 0.01).

Figure 2 represents the scratch test result 24 hours after the plasma or drug treatments. According to the results, in the case of the untreated cells, about 64% of the scratched area was covered by migrated cell in 24 hours; whereas, only 18% of the cells in the wound area grew within 24 h after plasma radiation (t(10) = 2.76, P < 0.01). With respect to the drug treatment, this amount reached 23% in comparison to untreated cells (t(10) = 2.76, P < 0.01).

### Plasma induces apoptosis in the cancer cells

**Figure 3:**
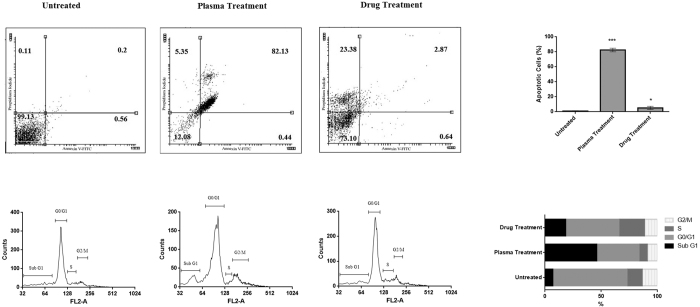
(**a**) Flow cytometry analysis of plasma and drug treated cells. The percentage of the cells in each region is indicated (***P < 0.001 and *P < 0.05). (**b**) Cell cycle analyses. The percentage of the cells in different phases of the cell cycle is indicated.

Figure 3a represents the ability of plasma to induce apoptosis in cancer cells based on flow cytometry results. This diagram shows that after plasma treatment, the cells migrate from the live zone to apoptotic zone. According to this diagram, more than 80% of the cells underwent apoptosis after plasma radiation (t(10) = 4.14, compared to the untreated group, P < 0.001). This amount was less than 3% for drug treatment (t(10) = 1.81, compared to the untreated group, P < 0.05). It is worth noting that in the drug treatment, cells underwent the necrosis (23.3%, t(10) = 2.76, P < 0.01). Figure 3b represents the cell cycle analysis after plasma radiation. These results indicate that half of the cells remained in SubG1 phase after plasma radiation (t(10) = 4.14, P < 0.001) while this amount was about 20% after drug treatment (t(10) = 2.76, P < 0.01). In addition less than 35% of the cells remain in the G0/G1 stage after the plasma treatment in comparison to the untreated samples (t(10) = 2.76, P < 0.01). This value reaches to 50% in the drug treated cells compared to the untreated samples (t(10) = 2.76, P < 0.01). Finally, there is no significant change in the population of the S phase cells in the case of plasma treatment and G2/M phase in both plasma and drug treatment compared to the untreated samples (P > 0.05).

### Plasma suppressed the tumor growth in the animals

**Figure 4:**
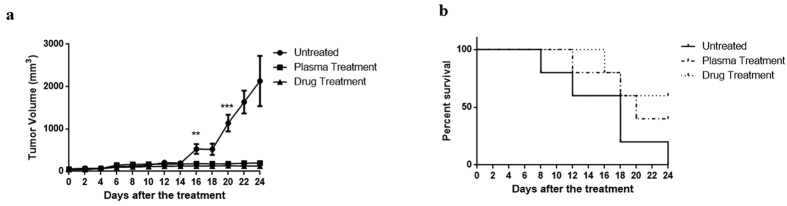
(**a**) Tumor volume measurements during the 24 days after plasma and drug treatments compared to the control group (***P < 0.001 and **P < 0.01). (**b**) Survival rate of the animals in 24 days after plasma and drug treatments.

Figure 4a represents the tumor size after plasma radiation at different days following the plasma treatment. This diagram shows no significant difference between the treated (by plasma and doxorubicin) and untreated groups up to 14 days after the treatment (P > 0.05), while after the day of the 16, the tumor suppression induced by plasma significantly differed from untreated sample (F(2,15) = 11.33, P < 0.001). The tumor size dramatically increased in the control group, insofar as it reached 500 mm^3^ on the 16th day (compared to day 0, P < 0.001) and almost 2000 mm^3^ after 24 days (compared to day 0, P < 0.001); whereas, 16 days after plasma radiation, tumor size did not significantly change as compared to the first day of treatment (P > 0.05). Drug treatment of the animals also significantly prevented the increase in tumor size (Compered to day 0, P > 0.05). Moreover, after the plasma treatment the survival rate of the animals was followed up for 24 days. Plasma treatment led to the survival of about 40% of the mice until the last day of observation; whereas, this amount was 60% in the drug treated group (Fig. 4b).

### Plasma affects the cells in the tumor area

**Figure 5:**
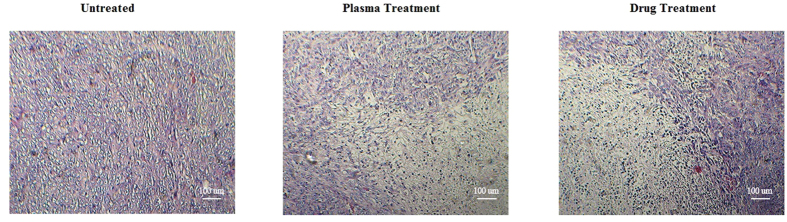
Hematoxylin and Eosin staining of the tumor tissue. The images show the depth of 2–3 mm of the tumor surface which clearly demonstrates the adverse effects of the drug and also the plasma on the cells.

To investigate histological differences between treated and control tumors, we performed H&E staining of the tumor sections (Fig. 5). Although control tumors exhibited a homogeneous distribution of viable cells, the sections of the plasma and drug treated tumors showed shrinkage of the cells, nuclear condensation and fragmentation which are indicative of apoptosis.

### Plasma induces apoptosis in the tumor cells

**Figure 6:**
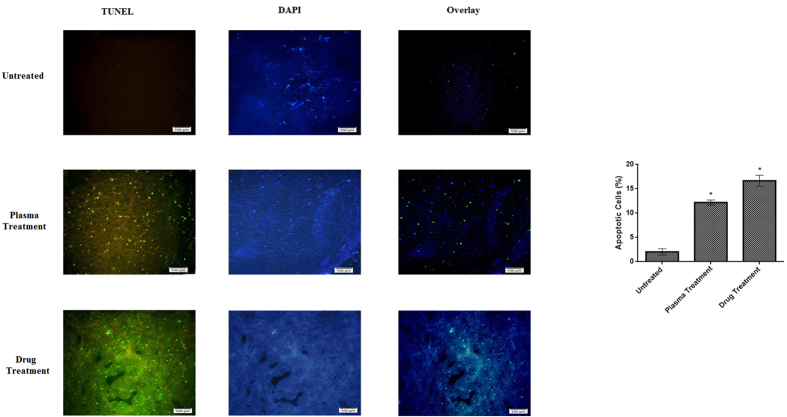
TUNEL assay for detection of apoptosis in the tumor tissue. The images show the depth of 2–3 mm of the tumor surface. Overlay images are made by merging of TUNEL and DAPI staining images. The scale of the images is 100 μm. The percentage of the apoptotic cells is compared to the untreated group (*P < 0.05).

Figure 6 indicates the presence of the apoptotic cells in tumor tissue 48 hours after the plasma treatment. Results of TUNEL assay suggested that about 13% of the cells underwent apoptosis on a cross-section of tumor tissue after plasma radiation (t(10) = 1.81, compared to the untreated group, P < 0.05). whereas, 17% of cells underwent apoptosis in drug treated group (t(10) = 1.81, compared to the untreated group, P < 0.05).

### Plasma increases P-53 level and Bax/Bcl-2 ratio

**Figure 7:**
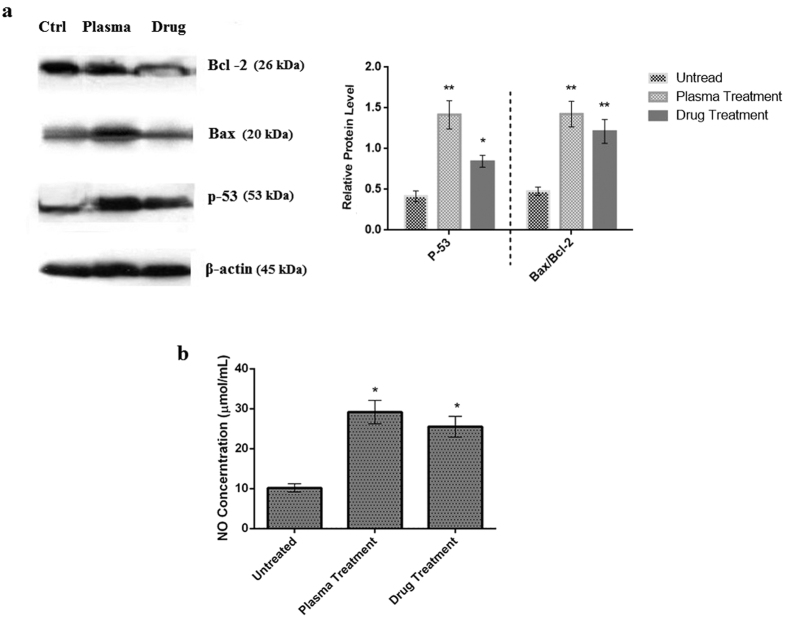
(**a**) Western blot analysis to determine the expression of Bax and Bcl–2 in extracts from tumor cells in each treatment group. Ctrl: Untreated group, Plasma: Plasma treated group, Drug: Doxorubicin treated group. Compared to the control group (**P < 0.01 and *P < 0.05). (**b**) Nitric oxide concentration in the blood of the animals (*) compared to the control group (*P < 0.05).

Figure 7a shows the expression of p-53, Bax, and Bcl-2 in tumor cells. Results suggest that plasma treatment significantly increased the expression of p-53 (Increment factor: 3.5, t(10) = 2.76, P < 0.01). The increase in p-53 expression in the case of drug treatment, compared to the untreated group, is also significant. (Increment factor: 2, t(10) = 1.81, P < 0.05).

Increase in the Bax/Bcl-2 ratio in tumor cells treated with plasma is another finding which is of great importance. This amount increases further in the plasma treatment group compared to the control group (Increment factor: 2.8, t(10) = 2.76, P < 0.01). The increment factor reaches to 2.4 for drug treatment group (t(10) = 2.76, P < 0.01).

### Nitric Oxide level increases after the plasma treatment in the blood of the animals

Figure 7b represents the amount of nitric oxide in blood of the mice. This diagram shows that the amount of nitric oxide in the blood increased three times in plasma treated group compared to the control group. This increase is 2.8 times in the case of doxorubicin treated group.

## Discussion

This study is among the initial advances in the use of cold atmospheric plasma inside the body of a living animal. *In-vitro* results showed that the plasma initially developed apoptosis in 4T1 cells (breast cancer cell line), and also inhibited the cell metastasis. The *in-vivo* study revealed that the μ-plasma treatment stopped the tumor growth. The findings also indicated that the plasma caused apoptosis in the tumor cells through the activation of the p-53 and Bax/Bcl-2 proteins.

By focusing on the cellular events, the results revealed that the plasma treatment could stop the cancer cells in Sub G1 phase, and could prevent them from entering into the DNA segmentation phase. Thus, more than 80% of the cancer cells which were treated with the plasma radiation underwent the apoptosis. This result is in agreement with the findings of many previous studies^1^,^3^,^31^,^32^. Partecke *et al*.^33^ observed that the argon plasma jet induced about 80% cell death which is comparable with the results of the present study. However, this study showed that the μ-plasma jet could lead to apoptotic death in the cancer cells.

Among all the *in vivo* plasma treatment studies, there are few reports which showed the design and optimization of the μ-plasma jet for utilizing in the patient’s body. One of the most important concerns about the μ-plasma jet is the temperature of the plasma jet plume, as the elevated temperature may cause the physical damage to the normal tissue. Zou *et al*.^28^ optimized the temperature of the μ-plasma jet by using the resistor and controlled the temperature of the plasma region. The low temperature of the plasma (less than 35 °C) in the present study associated by the high electron density could guarantee that the reactive species in the cold plasma could react with the tumor cells without any temperature hazard. The H&E staining showed that the plasma affected the tumor cells. In the analysis of the surface of the plasma treated tumors, the absence of the crust and of necrotic areas suggests little or no thermal impairment (data not shown) which is in conjunction with the results of *in vitro* experiments.

As mentioned earlier, few studies have been performed on the *in vivo* effects of plasma^33^,^34^,^35^. However, the majority of them have radiated the plasma from outside of the body. Van Damme *et al*.^36^ have shown that plasma exposure for 2 minute and 3 times in each treatment session by Dielectric Barrier Discharge (DBD) can decrease the size of glioma xerograph by 30%. The present research indicated that the *in-situ* plasma treatment could suppress the tumor growth until 24 days after the plasma treatment. In this regard, Kidar *et al*.^22^ showed the growth suppression of bladder (SCaBER) tumor by 5 minutes plasma treatment. They demonstrated that the plasma treatment could extend the life-span of the cancerous animals which is also in agreement with the results of this study. However, the death of animals in the plasma and drug treated group during the period of 24 days of observation, may be due to the other factors such as infection of the animals due to the low systemic immunity of the animal or the migration of the cancer cells to another organs^37^. Moreover, our results showed that the plasma treatment led to comparable results with those of the conventional chemotherapy drug treatment.

This study showed that the μ-plasma jet could inhibit the tumor growth in one plasma radiation session that lasted for 3 minutes. The differences in the effective treatment time between this study and other studies may be attributed to the differences in the sources, including the direct impact of the plasma ROS on the tumor as the most probable reason. When plasma is radiated from outside of the body, skin may act as a barrier that prevents such reactive and unstable species to reach the tumor tissue; whereas, in the μ-plasma method, these species can reach the tumor easily and trigger several chemical reactions, leading to a greater population of apoptotic cells. In addition, Collet *et al*.^38^ study showed an increase in the amount of nitric oxide in the blood after plasma treatment which is conjunction with our study. Yan *et al*.^39^ proved that the increase in the amount of the ROS and nitric oxide were correlated with the increase of the cell death by the lipid peroxidation in the cell membrane. The reactive species in the atmospheric plasma such as OH, NO, O, N_2_^+^ may cause the production of intracellular ROS^40^. The balance between the extent of the induced oxidative stress and the anti-oxidative mechanisms in the cells would render the cells toward death or survival^41^.

There are several methods which are now being in use for the cancer treatment such as chemotherapy^42^, surgery^43^ and radiotherapy^44^. The conventional methods have some disadvantages such as low rapidity and high cost of treatment. Moreover, adverse effects for both the operator and the patient are another disadvantages for such methods. While the plasma treatment may overcome the adverse effects of the traditional treatments, it should be noted that the plasma can be considered as a local treatment tool and does not exert the systemic therapeutic effects like chemical drugs. This seems to be a major issue for the plasma therapy for patients in clinical stage.

In summary, this study showed the effect of 250 micron non-thermal plasma jet inside the animal body on the suppression of the tumors growth. The *in-vitro* results showed that the μ-plasma could induce apoptosis in the 4T1 breast cancer cells and could inhibit the migration of the cells. The *in-vivo* results demonstrated that the μ-plasma suppressed the tumor growth and induced apoptosis in the tumor cells. The results of this study is a primary research for utilizing the atmospheric pressure plasma inside the patient’s body in the future.

## Material and Methods

### Ethics statement

All animal maintenance and procedures were in accordance with recommendations established by the Animal Ethics Committee of Tarbiat Modares University, Faculty of Medical Sciences as well as the United States NIH guidelines (publication no. 85-23). The protocols were approved by the Ethics Committee of Tarbiat Modares University, Faculty of Medical Sciences. All surgeries were performed under deep anesthesia, and all efforts were made to minimize suffering.

### Plasma setup and characterization

**Figure 8:**
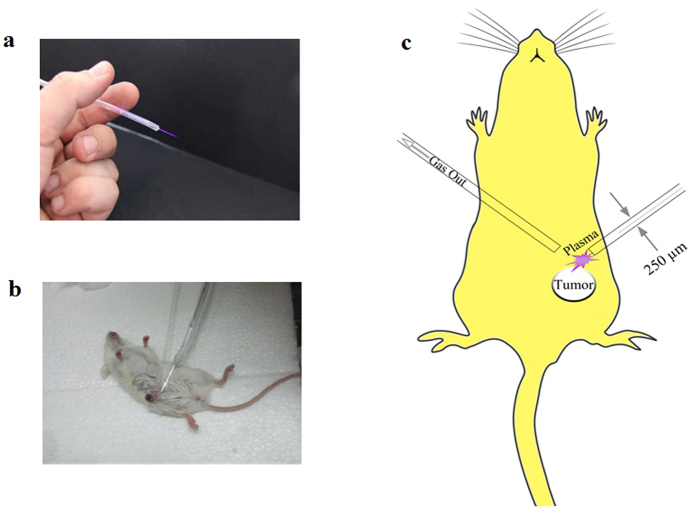
(**a**) Real discharge photo of μ-plasma jet. (**b**) Utilizing the μ-plasma jet inside the rat’s body. (**c**) Schematic of using μ-plasma jet inside the animal’s body.

The μ-plasma jet consisted of a 30 cm long and 3 mm thick Nelaton catheter. At the end of the catheter, a 250 μm capillary tube (length = 5 cm) were attached and insulated by epoxy. A copper wire were inserted into the catheter downward up to 2 cm from the capillary tube. The copper wire connected to the high voltage power supply. The power of the electrode was driven by a 6 KHz pulsed DC (Duty cycle = 20%) with the voltage of the 4 kV. The feeding gases for this study were 99.999% pure helium (He) which was injected into the catheter with 0.5 lit/min gas flow rate. The plasma plum exits from the capillary tube by the ionization of the gas (Fig. 8a).

For plasma characterization, first the emitted light from the plasma was collected by AvaSpec –ULS 2048 (Avantes Co, NL) spectrometer. The spectrum of the irradiated light was collected from 200–1100 nm with optical resolution of 0.1 nm. In order to evaluate the temperature of the ions and the electrons, the second positive system transition (370 nm to 382 nm) which obtained from the plasma spectrum was analyzed by SpecAir software. The temperatures of ions and electrons are figured out by equality of rotational and vibrational temperatures respectively. Finally the electron density of the plasma can be evaluated by analyzing the H_α_ transition at 658 nm by the procedure which has been discussed in the previous study^45^.

### Cell culture and plasma treatment

The mouse metastatic breast cancer 4T1 cells (ATCC CRL 2339, Pasteur institute, IR) were cultured in RPMI-1640 (Gibco Co, USA) supplemented with 10% (v/v) fetal bovine serum (FBS) (Gibco CO, USA), 100 U/ml penicillin and 100 μg/ml streptomycin (Sigma- Aldrich, USA) in a humidified incubator supplied with 5% (v/v) CO2 at 37 °C. Doxorubicin (Sigma-Aldrich, USA) at the concentration of 0.5 μg/ml as a conventional chemotherapy drug^46^ was employed as a potent apoptotic agent. The dosage of the drug was obtained from the previous studies^13^,^47^. The plasma was exposed to the cells for 1 minute and the distance between the cultured cells and the micro-plasma jet tip was set to 1 cm in all *in-vitro* experiments. Moreover, no significant changes in temperature were observed during the plasma treatment. All the *in-vitro* experiments were repeated for 3 times.

### Wound healing Assay

1 × 10^5^ cells/well of 4T1 were cultured in 6 well culture plates. After the cells attached as a monolayer and reached the confluency of 70–75%, the cells of each well were scratched and then treated with plasma for 1 min and examined after 24 h following the plasma treatment. The cell migration to the scratched area was examined with inverted microscope. In order to quantify the cell migration in the wound healing assay the ImageJ software was employed under the standard protocol^48^.

### Flow cytometry analysis

Apoptotic cells were quantified by annexin V-FITC and Propidium iodide (PI) double staining, using an annexin V-FITC apoptosis detection kit (Sigma-Aldrich, USA) according to manufacturer’s protocol. 5 × 10^4^ cells/well of 4T1 were seeded into 12 well flat plates. After the confluency of 70% was reached, the media was aspirated and the cells were washed with phosphate buffered saline (PBS), then the cells were treated with plasma and examined 24 h after the plasma treatment. Trypsinized 4T1 cells were collected and washed twice with PBS, centrifuged at 2000 RPM for 5 minutes and added 500 μl of the binding buffer. Then, 5 μl of Annexin V–FITC and 5 μl of PI were added and the cells were gently vortexed. Cells were then incubated for 15 minutes at room temperature (25 °C) in the dark. Finally, the cells were analyzed by flow cytometer (Becton Dickinson, USA).

### Cell cycle analysis

4T1 cells (1 × 10^5^ cells/well) were cultivated in 6 well plates. After reaching 70% of confluency, the media was removed, the cells were washed with PBS, treated with plasma and examined 36–38 h after the plasma treatment. Then the cells were harvested and washed by PBS and then 800 μl of cold 75% ethanol was added drop wisely to the cells while pipetting to minimize clumping of the cells. The cells were kept for15 minutes on ice. Ethanol-fixed cells were washed twice with PBS and then 300 μl of the solution containing 10 μ PI (Sigma-Aldrich, USA) (1 mg/ml), 1 μ triton X-100 (Sigma-Aldrich, USA), 20 μl RNase A (CinnaGen, IR) in 1 ml PBS, were added to the cells. Samples were incubated for 15 min at room temperature in the dark and were analyzed by flow cytometer (Becton Dickinson, USA).

### Animal study

1 × 10^6^ 4T1 cells in PBS were injected subcutaneously to 15 female BALB/c mice aged 4–6 weeks with the average weight of 20 ± 5 gr (Purchased from Pasteur institute, IR). Mice divided into three groups (control, treated with plasma, treated with doxorubicin) and each group consists of 5 mice. After the tumor size reached 5 ± 0.2 mm the skin of the animals of the first group, at a distance of 1.5 cm from the tumor edge, were drilled by sterile blade to make a hole with the diameter of about 0.5 mm. The plasma probe was injected subcutaneously through the hole. In addition, a capillary tube inserted subcutaneously to the other side of the tumor to evacuate the gas (Fig. 8b for real image and Fig. 8c for schematic presentation). The plasma was exposed to the tumor for 3 minutes at one session. In the drug treated group the mice were administered doxorubicin at the dosage of 5 mg/kg. The third group received no treatments. Tumor volumes were calculated by formula V = 0.52 × (X^2^Y) every two days and the study was followed for 24 days. It should be noted that all of the procedures were done under the full anesthesia by intra-peritoneal injection of ketamine (100 mg/kg) and xylazine (10 mg/kg) (Alpha Co., IR). It’s notable to state that the animals in the control group were also under anesthesia for the same period of time.

### TUNEL assay- Hematoxylin and Eosin staining

Apoptotic tumor cells were determined by terminal deoxynucleotidyl transferase- deoxyuridine triphosphate nick end labeling (TUNEL) staining (In Suit Cell Death Detection Kit, Fluorescein, Roche, CH). In this regard, 48 hours after the plasma treatment, half of the tumors were extracted from the animals 48 h after the plasma or drug treatment. The tumor was fixed in 4% formaldehyde in PBS for 24 h and embedded in paraffin for sectioning. The tumor sections were adhered to the glass slides pretreated with 0.01% aqueous solution of poly-L-lysine. The slides were heated at 60 °C followed by washing in xylene and rehydration through a graded series of ethanol. Finally the TUNEL staining of the samples were followed according to the manufacturer protocol and the slides were observed under the fluorescence microscopy and the images were processed by ImageJ software^49^. Moreover, Hematoxylin and eosin (H&E) staining were used to reveal the histopathological changes of the tissue.

### Western blotting

For western blotting analysis half of the tumors were collected from the animals. Extraction of proteins from samples were performed by RIPA buffer (10 mM Tris–HCl, pH 7.4, 150 mM NaCl, 5 mM EDTA, 1% Triton X-100, 0.1% sodium dodecyl sulfate and 0.5% Sodium deoxycholate) containing protease and phosphatase inhibitor cocktails. Protein concentrations were examined by Bradford assay^50^. Equal amounts of the total proteins were run on 12% Sodium dodecyl sulfate-polyacrylamide gel (SDS-PAGE assay) and transferred to the polyvinylidene difluoride (PVDF) and then probed with specific primary antibodies including rabbit polyclonal anti-Bax (1:200), mouse polyclonal anti-Bcl2 (1:200), rabbit polyclonal anti-P53 proteins (1:200) (Santa Cruz Biotechnology, USA) and secondary antibodies conjugated with horse radish peroxidase (HRP) (Cell Signaling Technology, USA). β-Actin was used as the control. Blots were developed by using DAB (Diaminobenzidine) staining.

### Nitric oxide (NO) concentration assay

The blood of the animals were collected 48 hours after the plasma treatment, and the plasma was obtained by centrifugation of the whole blood. The serum samples were stored at −70 °С until the NO assay was performed. Briefly, nitrite concentration was determined, using the standard Griess reagent by adding 50 μl of the test solution to 96-well flat-bottomed plates containing 50 μL of Griess reagent (Promega Co., USA). The absorbance of each well was measured by microplate reader (BioTeK, USA) at 540 nm and the nitrite concentration was determined by comparing the optical density at 540 nm (OD540) with a standard curve (1 to 200 μM) by the standard protocol^51^.

### Statistical analyses

Results were expressed as mean ± standard deviation (SD) of the wound area and calculated by GraphPad Prism Software. Student t-test was used for comparing the plasma and drug treated and untreated groups. In addition, one way-ANOVA test was employed to compare the mean of each group with that of the control mice in plasma and drug treatments for the tumor volume measurements and survival tests. In this regard, t (a) and F (a,b) refer to the critical t value and F distribution respectively. The F distribution is a right-skewed distribution and mostly employed in analysis of variance and critical t value is for estimating the mean of a normally distributed population in situations. These functions show the validity of the hypothesis in each comparison in addition to P value. It should be noted that, P-values < 0.05 (*) were considered as significant.

## Additional Information

**How to cite this article**: Mirpour, S. *et al*. Utilizing the micron sized non-thermal atmospheric pressure plasma inside the animal body for the tumor treatment application. *Sci. Rep.* **6**, 29048; doi: 10.1038/srep29048 (2016).
